# Alcohol consumption and hazardous drinking in western Kenya—a household survey in a health and demographic surveillance site

**DOI:** 10.1186/s12888-015-0603-x

**Published:** 2015-09-25

**Authors:** Rachel Jenkins, Caleb Othieno, Linnet Ongeri, David Kiima, Peter Sifuna, James Kingora, Raymond Omollo, Bernhards Ogutu

**Affiliations:** Health Services and Population Research Department, Institute of Psychiatry, Kings College London, de Crespigny Park, London, SE 5 8AF UK; Department of Psychiatry, University of Nairobi, Nairobi, Kenya; Kenya Medical Research Institute, Nairobi, Kenya; Ministry of Health, Nairobi, Kenya; Kombewa Health and Demographic Surveillance Site, Kombewa, Kenya; Kenya Medical Training College, Nairobi, Kenya

## Abstract

**Background:**

Alcohol use and hazardous drinking have been studied in school children and in urban areas of Kenya, but there has been no adult survey of these issues in a rural household population.

**Methods:**

This study reports the prevalence of alcohol consumption and hazardous drinking in a household survey of a demographic surveillance site in rural Kenya. Information collected included demographic characteristics, socio-economic factors, recent life events and perceived social support. Alcohol consumption was assessed by questions about quantity and frequency. The Alcohol Use Disorders Identification Test (AUDIT) measured hazardous alcohol use. The Clinical Interview Schedule- Revised assessed common mental disorder, and the Psychosis Screening Questionnaire indicated the presence of psychotic symptoms.

**Results:**

The study found that lifetime and current alcohol consumption were 10.8 % and 9.2 % respectively. Current alcohol consumption was significantly higher in men (OR 0.4, *p* < 0.001 for women) and in the self-employed (OR 1.8, *p* = 0.013), after adjustment for factors significant at the bivariate level.

Hazardous drinking was significantly higher in men (OR 0.3, *p* < 0.001 for women), people living in larger households (OR 1.8, *p* = 0.021), people who were single (OR 1.7, *p* = 0.093), and in those who are self-employed (OR 1.8, *p* = 0.036), after adjustment for factors significant at the bivariate level.

**Conclusion:**

This study suggests that alcohol consumption and hazardous drinking in the general population in a poor rural area in Nyanza Province is still relatively low. This represents an important public health educational opportunity to keep such rates low before increasing income and employment opportunities enable higher access to alcohol and other substances, and before the higher consumption found by studies on urban youth, especially neighbouring Kisumu town, spreads to the rural areas.

## Background

Alcohol represents a significant public health hazard for Africa as marketing strategies of alcohol focus increasingly on efforts to exploit the African market, and as incomes gradually increase [1]. Alcohol consumption is responsible for 4 % of global disability-adjusted life years [2, 3]. Alcohol use is thought to be a causal factor in 60 types of diseases and injury, a contributory factor in 200 other diseases as well as being associated with violence, suicide, child abuse and sickness absence from work [4, 5]. Hazardous alcohol use particularly impacts on physical and mental health outcomes, and is associated with economic disadvantage in both resource-rich [6, 7] and resource-poor [8, 9] countries. While there continues to be debate about whether light alcohol consumption is cardio-protective in western countries [10–13], such an effect was not found in two Indian studies [14, 15].

In sub-Saharan Africa, evidence indicates that alcohol consumption is increasing in many developing countries within the region [16, 17]. In much of Africa estimates of alcohol consumption rely on volume of sales which is misleading, because the industry is poorly regulated, and there are many illicit and counterfeit products, as evidenced for example by recent reports from Kenya [18].

The main determinants of alcohol related harm are volume of alcohol consumed and pattern of consumption [19]. Therefore, in order to inform public health prevention strategies, it is important to study risk factors for overall alcohol use as well as hazardous drinking. Despite the Western evidence on the contribution of alcohol consumption and hazardous drinking to morbidity and mortality, there is a dearth of epidemiological studies in low and middle income countries. In Kenya, there have been epidemiological studies of harmful use of alcohol in adults attending medical facilities [20], schools [21], and small scale surveys [22, 23]. There has also been a larger scale survey examining prevalence of alcohol use with sociodemographic factors including poverty, marital status, sex and age in western Kenya [24]. However there has been no household survey examining the relationship of alcohol use and hazardous drinking with not only sociodemographic factors but also psychosocial factors. Furthermore the Lo et al. study examined prevalence of getting drunk in the last month but did not use a specific tool to assess hazardous drinking [24]. This paper therefore used the opportunity of a wider epidemiological survey of psychiatric morbidity in a demographic surveillance site in rural Kenya to examine the prevalence of alcohol use and hazardous drinking, and their associated socio-demographic and psychosocial risk factors.

The area of the demographic surveillance site [25] is largely rural, with most residents living in villages, which are a loose conglomeration of family compounds near a garden plot and grazing land. The majority of the houses are mud-walled with either grass thatched or corrugated iron-sheet roofs. Water is sourced mainly from community wells, local streams and the lake for those living on the shores of Lake Victoria. Most water sources are not chlorinated. Subsistence farming, animal husbandry and fishing are the main economic activities in the area.

## Methods

Data for this analysis were drawn from a wider epidemiological household survey of psychiatric morbidity, immunity and malaria in a demographic surveillance site in rural Kenya to examine the prevalence of alcohol use and hazardous drinking, and their associated sociodemographic and psychosocial risk factors. The data for this study was collected between December 2012 and June 2013.

### Study population

The sample frame is a subdistrict in Kenya, in an area endemic for malaria, namely Maseno area within Kisumu County, Nyanza Province, Western Kenya which has a population of 70,805 [15]. Females constitute 53 % of the population. The mean household number is 4 people per household with a population density of about 374 people/km^2^. The population is largely young with a mean age of 23 years. The population 0–14 years constitutes 46 %, ages 15–64 years constitute 49 % and ages 65 + years constitute 5 %.

**Fig. 1:**
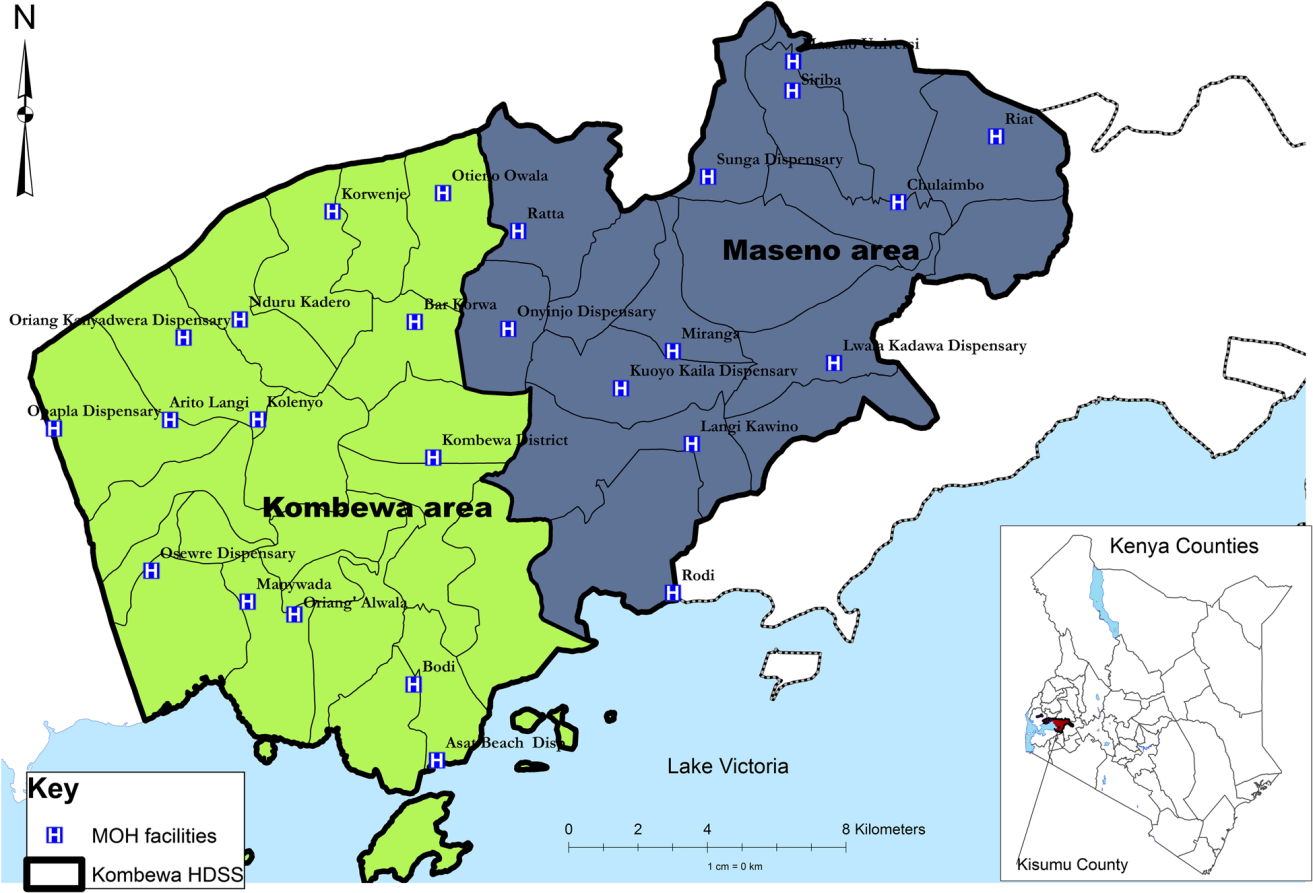
Location of study site

The population is primarily rural black African, and the languages spoken are Luo (predominant ethnic group), Kiswahili and English (Fig. 1).

The study sample was selected from Maseno Area within Kisumu County, western Kenya. Maseno Area is sub-divided into 4 locations, 17 sub-locations and 184 enumeration areas (villages) based on mapping work done earlier by the Kombewa Health and Demographic Surveillance System (Kombewa HDSS) run by the KEMRI/Walter Reed Project. The Kombewa HDSS is a longitudinal population registration system set up to monitor the evolving health and demographic problems of the study population in Kombewa and Maseno areas [25]. Some villages with less than 50 households were merged together to create new enumeration areas, so that the final total of enumeration areas was 170. A random sample of 7 households was drawn from each enumeration area, to give a projected sample of 1190 households, and hence 1190 adults. Village maps were used to assign households and guide the research assistants during the survey. Using the Kish Grid Method, one individual was selected at random from each of the sampled households [26]. Thus only one individual per household was interviewed. A total of 1190 households were visited, and a total sample of 1147 participants agreed to be interviewed. The demographics and reasons for the refusal were recorded in notebooks by the research assistants.

#### Study procedures

Meetings were held with community leaders to explain the purpose of the study and to answer questions. The participants in the survey were approached for informed consent, and then received a structured epidemiological assessment using the Clinical Interview Schedule –Revised, of mental disorders, accompanied by additional sections on socio-demographic data, life events, social networks, social supports, disability/activities of daily living, quality of life, use of health services, alcohol and substance abuse, adapted from the UK adult psychiatric morbidity schedule [27].

The interview was administered by one of a group of 20 research assistants using a PDA, on which the interview questions were programmed and responses were recorded. The research assistants received a 5 day training course, and were supervised in the field by a field manager. The survey was administered in either English or Swahili or Luo, depending on which language the participant found easiest. The research assistants were fluent in all three languages, and the questions were available on the PDA in all three languages. The research assistants all came from the local community, so had knowledge of the community and the social norms. Each session took between 1 and 2 h depending on the level of morbidity. The interviews were conducted in a secluded section of the respondent’s home if privacy was possible, or else in a quiet place outside the home which was well out of earshot of other people.

Information collected included demographic characteristics, socio-economic factors, recent life events and perceived social support. The Clinical Interview Schedule- Revised (CIS-R) [28], assessed common mental disorder (CMD), and the Alcohol Use Disorders Identification Test (AUDIT) [29], measured hazardous alcohol use.

Demographic information collected included sex, age, marital status, ethnicity, and household status (head, spouse or other). Socio-economic factors included employment status, income, education attainment, and housing variables. We did not collect data on religious affiliation.

Current alcohol use was determined by a positive response to the question:”*Do you ever drink alcohol nowadays, including drinks you brew or make at home?”* Lifetime non-drinkers (abstainers) were those who answered yes to *‘Have you always been a non-drinker’.* The category of lifetime drinkers included were those who had not always been a non-drinker and current drinkers.

The AUDIT is a cross-culturally validated instrument for assessment of alcohol misuse in the general population. The ten- item instrument includes questions to determine patterns of drinking considered harmful, hazardous and symptomatic of dependence in the preceding 12 months [29].

The CIS-R [28] is a gold standard instrument for use by lay interviewers in assessing common mental disorders in community settings, which has been widely used in low-income countries [30–32], including Kenya [33] and Tanzania [34, 35]. Scores are calculated from an average of four questions across 14 symptom types and taken together with algorithms based on the ICD-10 [36] provide six possible neurotic diagnoses including depressive episode (mild, moderate or severe), obsessive compulsive disorder, panic disorder, phobic disorder, generalised anxiety disorder and mixed anxiety/depressive disorder.

Respondents were given a list of 18 different stressful life events, and asked to say which, if any, they had experienced in the past six months. The list included relationship problems, employment, financial crises and victimisation experiences. The list was originally developed for the 1993 British psychiatric morbidity survey [37, 38] and tailored for the Tanzanian and Kenya contexts.

Perceived social support was assessed from respondents’ answers to seven questions previously used for the 1992 Health Survey for England [39], and the British Surveys of Psychiatric Morbidity [40, 41]. Participants responded “true”, “partly true” or “certainly true” in response to the question ‘There are people I know who’; (i) Do things to make me happy; (ii) Who make me feel loved; (iii) Who can be relied on no matter what happens; (iv) Who would see that I am taken care of if I needed to be; (v) Who accept me just as I am; (vi) Who make me feel an important part of their lives; and (vii) Who give me support and encouragement.

Information on social networks was obtained through questions about the number of friends or relatives who informants felt close to including (i) Adults who lived with the respondent and to whom they felt close; (ii) Relatives living elsewhere to whom they felt close; and (iii) Friends or acquaintances living elsewhere who informants would describe as close or good friends. These questions were taken from psychiatric morbidity surveys conducted in Britain [42, 43].

Specific questions were also asked about caring responsibilities (Do you give care due to long term physical or mental disorder or disability? And if yes, time spent giving care in a week); about growing up with one natural parent or two until age 16; and about spending time in an institution before the age of 16.

### Statistical analysis

We examined the prevalence of alcohol consumption and calculated the prevalence of hazardous drinking. We also examined the predictors of current alcohol consumption and hazardous alcohol consumption. The bivariate analysis calculated odds ratios (with 95 % confidence intervals) to quantify the level of association of each variable with current alcohol consumption and with hazardous alcohol use. We then conducted logistic regression to calculate adjusted odds ratios. Both bivariate analysis and adjusted analysis were conducted using STATA version 11.2 [44]. The level of statistical significance was set at 5 %. Logistic regression computes the relevant odds for each predictor or interaction term, takes the natural logarithm of the odds (computes the logit), conducts a linear regression analysis on the predicted values of the logit, and then takes the exponential function of the logit to compute the odds ratio. We assessed the goodness of fit as well as the model complexity using the Akaike’s information criterion (AIC) and the final model we ended up with is the one that maximized AIC. We did not employ other model selection techniques as we assumed a linear model with errors normally distributed.

Households have been categorized into different socio-economic levels using an index of household assets, constructed applying the principal component analysis procedure, as a proxy indicator for socio-economic status. In developing the asset quintiles, type of house, roofing & walling material, source of water, toilet facility and land have been used [45, 46]. Each question in the AUDIT is scored between zero and four with a score of eight and over considered indicative of hazardous use. A score of 12 or more across the 14 sections of the CIS-R was considered an indication of any CMD [28, 37, 38]. Life event scores were grouped into none, one, two, and three or more life events. Perceived social support scores were categorised into no, moderate or severe lack of social support. Social network scores were grouped into “none to three”, “four to eight” and “nine or more”.

### Ethics

Ethical approval was granted by the Kings College London and Kenya Medical Research Institute Boards of Research Ethics respectively (PNM/11/12-54, SSC2374), and permission was obtained to conduct the study in households in Maseno area, which is part of the KEMRI/WRP Kombewa HDSS. Consent to participate in the study was voluntary and was administered at individual level after receiving consent from the head of household. Head of household was defined as the father when he was there and if he was not there then it was the mother, and if neither were present it was the grandfather or grandmother. This was in keeping with the cultural norms in the area. Written and witnessed informed consent was asked of the participants to take part in the study.

## Results

Table 1 gives the life time and current prevalence rates for alcohol consumption and hazardous drinking in men and women. 14.5 % of men and 6.8 % of women reported life time drinking, and slightly lower proportions reported current alcohol consumption (13.1 % of men and 4.8 % of women). Hazardous drinking was reported by 9.5 % of men and 2.9 % of women.

**Table 1:** Life time and current prevalence rates for alcohol, tobacco and cannabis use in Maseno area, Kenya

	Life time alcohol	Current alcohol	Hazardous drinking
		N(%)	N(%)
Total Prevalence	124 (10.8)	105 (9.2)	73(6.4)
Male prevalence	87 (14.5)	79 (13.1)	57(9.5)
Female Prevalence	37 (6.8)	26 (4.8)	16(2.9)

Table 2 examines the sociodemographic, economic, social and psychological risk factors for current alcohol use.

**Table 2:** Prevalence of current alcohol use and its relationship with socio demographic, economic, social variables and CMD, using bivariate analysis (odds ratios)

Factors		Prevalence: n (%)	Unadjusted OR	*p*-value
Sex	Male	79 (13.1)	1	-
	Female	26 (4.8)	0.3 (0.21 to 0.53)	<0.001
Age group	<30 years	32 (11.4)	1	-
	30-60 years	44 (9.8)	0.8 (0.52 to 1.37)	0.495
	>60 years	11 (6.4)	0.5 (0.26 to 1.09)	0.086
Household size	<=6 people	47 (8.3)	1	-
	>6 people	58 (10.0)	1.5 (0.97 to 2.18)	0.068
Marital Status	Married/cohabiting	66 (9.2)	1	-
	Single	22 (12.0)	1.3 (0.80 to 2.24)	0.250
	Widowed/divorced	17 (6.9)	0.7 (0.42 to 1.26)	0.416
Education	None	15 (11.5)	1	-
	Primary	40 (6.4)	0.5 (0.28 to 0.99)	0.046
	Secondary	38 (11.9)	1.0 (0.55 to 1.97)	0.899
	Post secondary	12 (16.9)	1.6 (0.69 to 3.58)	0.280
Employment status	Unemployed	35 (6.2)	1	-
	Self employed	58 (12.0)	2.0 (1.32 to 3.18)	0.001
	Employed	12 (12.1)	2.1 (1.04 to 4.16)	0.038
Asset Groups	Lowest, Q1	38 (9.6)	1	-
	Q2	39 (9.7)	1.0 (0.63 to 1.62)	0.969
	Highest, Q3	28 (8.0)	0.8 (0.49 to 1.35)	0.418
Perceived lack of social support	No lack: 0	1 (33.3)	1	-
	Moderate lack: 1-7	47 (15.0)	0.9 (0.57 to 1.44)	0.679
	Severe lack: 8+	71 (8.6)	-	-
Total Social Group size	3 or less	15 (10.4)	1	-
	4-8	50 (9.6)	0.9 (0.50 to 1.69)	0.780
	9 or more	40 (8.2)	0.8 (0.42 to 1.46)	0.436
Life events	0-1	36 (10.0)	1	-
	2-3	42 (8.8)	0.9 (0.55 to 1.39)	0.565
	4 or more	27 (8.7)	0.9 (0.51 to 1.46)	0.586
Presence of CMD	No	96 (9.3)	1	-
	Yes	9 (7.6)	0.8 (0.39 to 1.62)	0.526
Carer for more than 4 h*	No	2 (7.7)	1	-
	Yes	13 (7.6)	1.0 (0.21 to 4.64)	0.987
Spent time in institution before age 16	No	81 (8.8)	1	-
	Yes	24 (10.9)	1.3 (0.78 to 2.05)	0.333
Did not live continuously with both natural parents until age 16	No	93 (9.7)	1	-
	Yes	12 (6.8)	0.7 (0.37 to 1.28)	0.235

Factors significant at the bivariate level included an increased risk of alcohol use in men (OR 0.3, 95 % C.I. = 0.21 to 0.53, *p* < 0.001 for women), those with no education (OR 0.5, 95 % C.I. = 0.28 to 0.99, *p* = 0.046 for primary education) and those who are employed (OR 2.1, C.I. = 1.04 TO 1.35, *p* = 0.038) or self-employed (OR 2.0,C.I. = 1.32 to 3.18, *p* < 0.001).

Forward stepwise regression modelling allowed for adjustment of variables significant at the bivariate level (see Table 3). Current alcohol use remained significantly higher in men (OR 0.4, C.I. = 0.23 to 2.75, *p* < 0.001 for women) and the self-employed (OR 1.8, C.I. = 1.13 to 2.75, *p* = 0.013) .

**Table 3:** Risk factors for current alcohol use using logistic regression analysis (adjusted odds ratios)

Factors		Adjusted OR (95 % C.I)	*p*-value
Sex (=female)		0.4 (0.23 to 2.75)	<0.001
Employment status	Self employed	1.8 (1.13 to 2.75)	0.013
	Employed	1.5 (0.73 to 3.00)	0.283

Table 4 shows the relationship of hazardous alcohol use, (audit score of 8 or more), with sociodemographic variables, social variables and CMD. The prevalence of hazardous alcohol use (AUDIT score greater than 8) was 6.4 %.

**Table 4:** Prevalence of hazardous alcohol consumption and its relationship with socio demographic variables, social variables and CMD, using unadjusted odds ratios

Factors		Prevalence: n (%)	Unadjusted OR (95 % C.I)	*P* - value
Hazardous alcohol use		73 (6.4)		
Sex	Male	57 (9.5)	1	-
	Female	16 (2.9)	0.3 (0.16 to 0.51)	<0.001
Age group	<30 years	23 (8.2)	1	-
	30-60 years	29 (6.5)	0.8 (0.44 to 1.37)	0.379
	>60 years	10 (5.9)	0.7 (0.32 to 1.50)	0.356
Household size	<=6 people	25 (4.4)	1	-
	>6 people	48 (8.3)	2.0 (1.19 to 3.22)	0.008
Marital Status	Married/cohabiting	42 (5.9)	1	-
	Single	19 (10.4)	1.9 (1.05 to 3.28)	0.033
	Widowed/divorced	12 (4.8)	0.8 (0.42 to 1.57)	0.542
Education	None	11 (8.4)	1	-
	Primary	27 (4.3)	0.5 (0.24 to 1.02)	0.057
	Secondary	28 (8.8)	1.0 (0.50 to 2.17)	0.904
	Post secondary	7 (9.9)	1.2 (0.44 to 3.23)	0.728
Employment status	Unemployed	25 (4.4)	1	-
	Self employed	42 (8.7)	2.0 (1.22 to 3.40)	0.006
	Employed	6 (6.1)	1.4 (0.55 to 3.48)	0.483
Asset Groups	Lowest, Q1	27 (6.9)	1	-
	Q2	20 (4.1)	0.7 (0.38 to 1.25)	0.226
	Highest, Q3	26 (7.4)	1.1 (0.61 to 1.86)	0.838
Presence of CMD	No	65 (6.3)	1	-
	Yes	8 (6.7)	1.1 (0.50 to 2.28)	0.866
Social support group size	0-3	6 (4.2)	1	-
	4-8	35 (6.7)	1.7 (0.69 to 4.04)	0.261
	9+	32 (6.7)	1.6 (0.67 to 4.00)	0.278
Life events	0-1	28 (7.8)	1	-
	2-3	28 (5.9)	0.7 (0.43 to 1.28)	0.280
	4+	28 (5.5)	0.7 (0.37 to 1.29)	0.247
Perceived lack of social support	No lack: 0	0 (−)	1	-
	Moderate lack: 1-7	24 (7.7)	1.3 (0.80 to 2.19)	0.282
	Severe lack: 8+	49 (5.9)	-	-
	Positive	14 (5.2)	0.7 (0.38 to 1.30)	0.263
Carer for more than 4 h a week	No	1 (3.9)	1	-
	Yes	9 (5.3)	1.4 (0.17 to 11.44)	0.760
Spending time in institution before age 16	No	61 (6.6)	1	-
	Yes	12 (5.5)	0.8 (0.43 to 1.54)	0.523
Not living continuously with both natural parents up to l age 16	No	67 (7.0)	1	-
	Yes	6 (3.4)	0.5 (0.20 to 1.11)	0.084

Factors significant at the bivariate level included an increased risk of hazardous drinking in men (OR 0.3, C.I. = 0.16 to 0.51, *p* < 0.001 for women), those with no education (OR 0.5, C.I. = 0.24 to 1.02, *p* = 0.057 for those with primary education), those living in large households (OR 2.0, C.I. = 1.19 to 3.22, *p* = 0.008), those who are single (OR 1.92.0, C.I. 1.05 to 3.28, *p* = 0.033), and those who are self-employed (OR 2.0, C.I. = 1.22 to 3.40, *p* = 0.006) Table 5.

**Table 5:** Risk factors for hazardous drinking using logistic regression analysis (adjusted odds ratios).s.

Factors		Adjusted OR (95 % C.I)	*p*-value
Sex (=female)		0.3 (0.17 to 0.58)	<0.001
Household size (>6 people)		1.8 (1.09 to 2.97)	0.021
Marital Status	Single	1.7 (0.92 to 3.04)	0.093
	Widowed/divorced	1.2 (0.57 to 2.39)	0.671
Employment status	Self employed	1.8 (1.04 to 2.99)	0.036
	Employed	1.1 (0.42 to 2.74)	0.892

Table 5 shows the final adjusted model. The risk of hazardous drinking was increased in men (OR 0.3, C.I. = 0.17 to 0.58*p* < 0.001 for women), people living in larger households (OR 1.8, C.I. = 1.09 to 2.97, *p* = 0.021), people who were single (OR 1.7,C.I. = 0.92 to 3.04, *p* = 0.093), and those who are self-employed (OR 1.8, C.I. = 1.04 to 2.99, *p* = 0.036).

## Discussion

### Overall findings

This study reports the prevalence of current and life time alcohol use and hazardous drinking in a health and demographic surveillance site in a rural area of Kenya, near Lake Victoria, and found that current alcohol consumption was 9.2 %, with lifetime use only marginally higher at 10.8 %. The rate of hazardous drinking was 6.4 %. Rates were higher in men than in women. Risk factors were further explored for current alcohol consumption and for hazardous drinking. Current alcohol use was significantly higher in men (OR 0.4, *p* < 0.001 for women) and in the self-employed (OR 1.8, *p* = 0.013) in the adjusted analysis. Hazardous drinking was also greatly increased in men (OR 0.3, *p* < 0.001 for women), in people living in larger households (OR 1.8, *p* = 0.021), people who were single (OR 1.7, *p* = 0.093), and those who are self-employed (OR 1.8, *p* = 0.036). Contrary to expectation, psychosocial variables were not significant risk factors in the adjusted analyses.

### Comparison of findings with other relevant studies

The rates of alcohol consumption and hazardous drinking found in this household study of a rural district in western Kenya are relatively low compared to those found in other household studies of Kenya.

A 1990 cross-sectional survey of 15 324 household heads in Kisumu district in Kenya (thus including Kisumu town and the surrounding rural areas including our study area of Maseno, revealed that the reported rate of current alcohol use was 6.4 % [47], while a more recent survey near Kisumu of 72, 292 adults found a past month prevalence of 7.3 % [24], so it looks as if over an intervening quarter century the rate of alcohol use in this area of Kenya has only marginally increased.

In the rest of Kenya, reported rates are higher. Thus the World health Survey data for Kenya found a life time prevalence of alcohol consumption of 26.2 % for rural adults [48], and a national survey In Kenya [22] found a lifetime prevalence rate for alcohol of 39 % and a current prevalence rate of 13 % for people aged between 15 to 64 years. A later subnational survey of 500 households and 3500 adult respondents in Central Province [23] found a lifetime prevalence of 29.6 % (53 % in males and 8 % in females); and a current prevalence of 18 % (34 % in males and 3 % in females).

Studies of school children and college students tend to find still higher rates of alcohol consumption and hazardous drinking. A recent study of alcohol consumption in a sample of Kenyan secondary school students in 2011 found that nearly half (48.9 %) indicated past consumption of alcohol, with one fifth (18.5 %) maintaining usage [49]. Respondents from private schools had the highest proportion of current consumers at 22.9 %). Alcohol use was higher in males than females, in school grade 4 compared to grade 1, and was associated with increased sexual activity and violence.

Similarly, another survey of secondary school children, this time in Kisumu town, found that 57.9 % had consumed alcohol at least once in their life time, with rates higher in older age groups and in boys [50], while a much earlier survey of Nairobi school children conducted over 30 years ago found even then that around 10 % of students drank more than three times a week [51]. Since half the population in our study area is aged under 15, these young people represent a crucial population at future risk of sustained alcohol consumption and hazardous drinking. It is worth noting that in our study the proportion of hazardous drinkers is around two thirds of the men and half of the women who drank at least one unit in the last week, whereas the equivalent proportion in England [27] is half of the men and one third of the women. This may suggest that the trajectory to hazardous drinking is faster in Kenya than in England, and further research is needed to elucidate this.

Alcohol consumption is usually found to be higher in people with lower socioeconomic status [52, 53], and indeed a household survey of districts adjacent to our study area of Maseno found that the poorest had significantly higher rates of current alcohol consumption [24]. A study in two urban areas of Tanzania of differing levels of poverty, using similar methodology and instruments to this survey, found that in a random sample of 899 adults aged 15–59, rates of alcohol consumption were 17.2 %. Living in the less affluent area was associated with higher lifetime rates of alcohol use [53]. It is therefore surprising that we did not find an association with socioeconomic status or poverty in this study, although we did with self-employment. We also found a high proportion of abstainers who have never consumed alcohol and this phenomenon has been noted by others in Africa [54].

It is possible that the low rates of current alcohol consumption and of hazardous drinking found in this study result from a combination of the extreme poverty of the area such that it is very hard to pay for alcohol, the widespread religious conservatism of a high proportion of the population, and the potential effectiveness of the recent Kenya Alcoholic Drinks Control Act 2010, which is intended to control production, limit outlets, and control consumption and age of use [23]. In an area characterised by chronic poverty and disease, religion thrives giving hope to people struggling with a relatively harsh existence. We did not enquire about religious affiliation in our study but Maseno area has a relatively high proportion of conservative Protestant based Christian groups who are very strict about banning alcohol consumption e.g. Seventh Day Adventists and Legio Maria. The proximity of our study area to Kisumu town means that law enforcement against illegal brews is stricter than in more remote rural areas. Further studies are needed to clarify these possible influences.

Relationships between psychosocial variables such as life events and social supports and alcohol use and hazardous drinking are variable in the literature [57]. Studies in western countries have found that life events contribute to problem drinking while social support can buffer its effects [58], although the buffering effect of social support disappears in poor populations [59]. Relationships of alcohol with CMD are also variable. For example, the British national psychiatric morbidity survey did not find a relationship between alcohol dependence and CMD [27], but a relationship between hazardous drinking and CMD was found in Tanzania [53].

### Strengths of study

The strengths of the study are the use of a health and demographic surveillance site for the random sample of households, the high response rate, and the systematic approach to the clinical and sociodemographic assessments. The population in the surveillance site is regularly monitored by field staff who visit each household bi-annually to capture health and demographic information (Birth rates, Death rates, Causes of Death, Pregnancies, Immunization status, in-and out-migrations). Various studies nested on the DSS platform take advantage of the sampling frame inherent in the HDSS, whether at individual, household/compound or regional levels. This familiarity with survey procedures is likely to have been influential in the achievement of a high response rate.

### Limitations of study

As always, the potential for measurement error when using screening instruments should be acknowledged, given self-reported experiences may be subject to recall or social desirability or cultural response bias [55]. In addition, the measurement of alcohol units in Africa is difficult because of the ubiquity of relatively strong home brews [56] hence some bias due to measurement error may exist.

The implementation of the study was hampered by a number of logistical challenges which included the difficult terrain, posing problems for local transport for research staff, and continuing administrative difficulties, which led to delays in the implementation of the project. The interviewing period, initially planned to last 3 months, took place over a period of 6 months, and was temporarily halted for several weeks over the period of the 2013 election due to further fears of election unrest.

## Conclusion

The rates of current alcohol use and hazardous drinking found in this study of a poor rural area in Kenya remain relatively low, and represent an important public health prevention and educational opportunity to keep such rates low in this area before increasing income and employment opportunities enable higher access to alcohol, and higher rates of consumption with its associated health hazards, and before the higher consumption found by other studies in urban youth, especially neighbouring Kisumu town, spreads to the rural areas. We recommend that school health programmes and adult public health education strategies include information about the harmful effects of alcohol use and hazardous drinking, that access to cheap illegal brews continues to be restricted, that pricing policies of legal brews do not encourage cheaper availability of alcohol, that primary care staff are trained in early detection and prompt management of moderate and severe levels of alcohol consumption and hazardous drinking with its associated suicidal risk and physical comorbidity, as well as advice to those who drink regularly at low levels. We recommend that further research is conducted to elucidate the relationship between alcohol consumption, hazardous drinking and psychosocial variables.

## Abbreviations

CIS-R: Clinical interview schedule revised
CMD: Common mental disorders
PSQ: Psychosis screening questionnaire
AUDIT: Alcohol use disorders identification test
KCL: Kings College London
KEMRI: Kenya Medical Research Institute
KWHDSS: Kombewa West Health and Demographic Surveillance Site
